# Use of Vortioxetine in the Treatment of Major Depressive Disorder and Post-Traumatic Stress Disorder: A Case of Functional Recovery

**DOI:** 10.1192/j.eurpsy.2025.1942

**Published:** 2025-08-26

**Authors:** R. Albillos Pérez, M. Fernández Rodríguez, S. Castelao Almodóvar, P. Sanz Sánchez, R. González Núñez, C. Alguacil Núñez, A. Castelao Escobar

**Affiliations:** 1 Psiquiatría, Hospital Universitario Puerta de Hierro Majadahonda; 2 Psiquiatría, Hospital Universitario El Escorial, Madrid, Spain

## Abstract

**Introduction:**

Vortioxetine is an antidepressant with multimodal action that exerts its numerous effects by modulating neurotransmission in multiple systems, achieving not only antidepressant effects but also procognitive ones, along with an appropriate tolerability profile. Currently, vortioxetine is only approved for the treatment of major depressive disorder (MDD) in adults. There is currently no unified consensus on the most appropriate pharmacotherapy for post-traumatic stress disorder (PTSD), so this clinical case raises the possibility of using drugs with multimodal action, such as vortioxetine, in patients with comorbid PTSD and MDD.

**Objectives:**

To describe a clinical case of comorbid PTSD with MDD, highlighting the use of drugs with multimodal action.

**Methods:**

A 26-year-old woman began follow-up for an 8-month history of depressive mood, with apathy, anhedonia, and constant rumination about a significant life event, impacting her basic activities of daily living, along with death-related thoughts and fear of acting on them. She also presented with highly limiting tics, difficulty concentrating, mental blocks, slowed thinking, and mixed insomnia. The patient directly relates these symptoms to a traumatic event during her adolescence, which involved sexual abuse and threats during a conflicted romantic relationship. Over the years, she has experienced sequelae related to the traumatic event, such as avoidance behaviors regarding sexual matters, flashbacks with dissociative episodes during periods of heightened anxiety, difficulties in social relationships, and feelings of guilt, fear, and disgust. Treatment with Vortioxetine was prescribed, with progressively increasing doses over the following consultations until reaching 20 mg/day. After several visits to the emergency department during this time, the patient was referred to a psychiatric day hospital.

**Results:**

Thanks to this combined therapeutic plan, the patient has shown significant functional recovery, with marked improvement in the affective domain, regaining interest and enjoyment; and in the cognitive domain, demonstrating an increase in her ability to concentrate and in processing speed. Additionally, she has been able to maintain adequate sexual activity during this time.

**Conclusions:**

Antidepressants with serotonergic action present sexual side effects, so treatment must be individualized in patients for whom this area is particularly relevant, considering drugs with a lower rate of side effects in this regard. The complete functional recovery of MDD includes cognitive symptoms as one of the primary therapeutic objectives, so it is important to consider using drugs that are effective in improving the affected cognitive domains. Post-traumatic stress disorder could benefit from the use of drugs with multimodal action, although more studies are needed in the future.

**Disclosure of Interest:**

None Declared

